# Narcolepsy and emotional experience: a review of the literature

**DOI:** 10.1186/s12993-018-0151-x

**Published:** 2018-12-26

**Authors:** C. Schiappa, S. Scarpelli, A. D’Atri, M. Gorgoni, Luigi De Gennaro

**Affiliations:** Department of Psychology, University of Rome “Sapienza”, Via dei Marsi, 78, 00185 Rome, Italy

**Keywords:** Narcolepsy, Cataplexy, Emotions, Dreaming, REM sleep

## Abstract

Narcolepsy is a chronic sleep disorder characterized by excessive daytime sleepiness, cataplexy, hypnagogic hallucinations, and sleep paralysis. This disease affects significantly the overall patient functioning, interfering with social, work, and affective life. Some symptoms of narcolepsy depend on emotional stimuli; for instance, cataplectic attacks can be triggered by emotional inputs such as laughing, joking, a pleasant surprise, and also anger. Neurophysiological and neurochemical findings suggest the involvement of emotional brain circuits in the physiopathology of cataplexy, which seems to depending on the dysfunctional interplay between the hypothalamus and the amygdala associated with an alteration of hypocretin levels. Furthermore, behavioral studies suggest an impairment of emotions processing in narcolepsy-cataplexy (NC), like a probable coping strategy to avoid or reduce the frequency of cataplexy attacks. Consistently, NC patients seem to use coping strategies even during their sleep, avoiding unpleasant mental sleep activity through lucid dreaming. Interestingly, NC patients, even during sleep, have a different emotional experience than healthy subjects, with more vivid, bizarre, and frightening dreams. Notwithstanding this evidence, the relationship between emotion and narcolepsy is poorly investigated. This review aims to provide a synthesis of behavioral, neurophysiological, and neurochemical evidence to discuss the complex relationship between NC and emotional experience and to direct future research.

## Introduction

Narcolepsy is a chronic debilitating sleep disorder affecting humans and animals, characterized by excessive daytime sleepiness (EDS), cataplexy, hypnagogic hallucinations, and sleep paralysis. EDS consists of daily periods of an irrepressible need to sleep that leads to real sleep attacks, and is exacerbated by periods of relative physical inactivity (e.g., watching television, driving car, etc.). Also, the premature occurrence of REM sleep onset (SOREMs) characterizes these sleep episodes [1, 2], representing an essential element for diagnosing narcolepsy [3]. It should be underlined that unlike EDS, that may also occur in other disorders, cataplexy is a pathognomonic symptom of narcolepsy. It consists of a sudden drop in muscle tone, total or partial, triggered by intense emotions, especially those leadind to laughters, but also excitement and anger [4–9]. These attacks can last from a few seconds to a few minutes, during which the patient remains conscious but unable to move. These symptoms are necessary to diagnose narcolepsy [3], while hypnagogic hallucinations and sleep paralysis are considered auxiliary symptoms. Hypnagogic hallucinations are dream-like hallucinations that occur at sleep onset and can be very scary because the patient is not aware of their hallucinatory nature. Sleep paralysis, often associated with hallucinations, is an inability to move limbs, head, or to breathe normally, and can be frightening and even terrifying, especially the first time.

In the past, narcolepsy has been classified in two sub-types: narcolepsy with and without cataplexy [10]. This symptom-based diagnosis has become unconvincing due to the discovery of the role of hypocretin/orexin in cataplexy (hereafter referred to as “hypocretin”). The narcolepsy with cataplexy (NC) is associated with a low concentration of hypocretin in cerebrospinal fluid (CSF), while a normal hypocretin concentration in CSF appears to characterize the narcolepsy without cataplexy (N) [11–13]. Nevertheless, a small population of patients with hypocretin deficiency does not manifest cataplexy at the time of diagnosis [14, 15]. Considering the involvement of hypocretin in this sleep disorder, ICSD-3 [3] changed the terminology to “narcolepsy type 1” (hypocretin deficiency) and “narcolepsy type 2” (no hypocretin deficiency).

It should be noted that narcolepsy was described for the first time at the end of the nineteenth century [16–18], and since the beginning some authors focused on the relationship between emotional factors and narcolepsy, especially within the psychoanalytic framework [19]. Psychoanalysis and the first studies on narcolepsy were almost contemporary. Freudian theories on dreams led to a psychoanalytic explanation of this sleep disorder [20]. Interestingly, in this framework, sleep was considered a temporary escape from reality, and narcolepsy, with its unusual symptoms, was interpreted as related to some psychological condition. For instance, cataplectic attacks are often triggered by emotions, while frightening contents often characterize sleep paralysis and hallucinations. Also, often the symptoms first appear during the psychologically and sexually turbulent years of adolescence. In the light of above, it was hypothesized that narcolepsy could be considered a defense against anxiety and uneasiness associated with difficult personal issues. Some authors also investigated the clinical and personal stories of these patients, confirming the hypothesis of a link with emotional issues [e.g., 21]. However, these theories suggested sexual deviations and psychopathic personality structures as the underlying factors of narcolepsy [22, 23]. An involuntary consequence of this view was that narcoleptic patients might develop a sense of guilt over their symptoms.

At the end of the 1990s, the deficit in hypothalamic hypocretin neurotransmission as the main determinant of narcolepsy was discovered, and this disorder was established as an organic brain disease [24–26]. Narcolepsy has long been described as characterized by high co-morbidity for psychiatric disorders [27], often making the diagnosis difficult and delaying it. Nevertheless, it is still unclear if psychiatric symptoms are the result of the chronic disabling nature of the disease, or a “shared pathophysiology”, or a combination of both [28]. The current literature revealed that a high percentage of narcoleptic patients suffer from depression (57%) [29–31]. This co-morbidity may be due to the significant overlap in symptoms [31–33]. However, purely depressive symptoms like anhedonia, pathological guilt, and crying also occur in patients with narcolepsy [31]. Therefore, shared pathophysiology related to a hypocretin deficiency should be considered. For instance, lowered levels of hypocretin in post-mortem histopathological studies were found in depressed patients who completed suicide [34], but these findings have been inconsistent in other studies [35]. Also, the anxiety disorder has been reported in the 53% of patients with narcolepsy [32], but available studies are still relatively scarce. Lastly, comorbid schizophrenia and narcolepsy has been found, but the relationship between these diseases is controversial and potentially misleading because of the significant overlap of the hallucinatory phenomena [36–39]. The occurrence of psychotic and hallucinatory symptoms in narcolepsy has been reported as responsible for delayed diagnosis due to a misdiagnosis of schizophrenia [40–44]. In this respect, recent studies found that the comorbidity appears to be rare, underlying that further investigations should be carried out to improve diagnostic accuracy [45–47].

Although psychoanalytic theories proposed extreme etiological theories, and some symptoms of narcolepsy do depend on emotional stimuli, the relationship between emotion and narcolepsy is poorly investigated. On a general level, several studies examined the interaction between sleep and emotional regulation [for a review, 48], and sleep deprivation protocols demonstrated the association between sleep loss and daytime negative emotional consequences [e.g., 48–52]. Poor sleep quality is linked to increased negative emotions and decreased positive emotions [53–55], with patients suffering from insomnia showing negative mood more than good sleepers, especially in the evening [56]. Idiopathic nightmares are linked to psychological distress [57] and an alexithymia personality style [58]. Anxiety may increase the occurrence of somnambulism [59].

The link between sleep and emotions also emerges in the field of clinical psychiatry. It is difficult to identify any mental health condition with symptoms related to mood or emotion in which sleep disturbance is not listed as a formal symptom or a common feature of the condition [60], such as in major depression and post-traumatic stress disorder (PTSD).

To sum up, it could be stated that in narcolepsy the relationship between sleep and emotional factors is particularly robust, mainly because the symptoms seem to be exacerbated by emotional states. In this review, we discuss the complex relationship between NC and emotional experience, providing a synthesis of behavioral, neurophysiological and neurochemical evidence.

## Methodological note

Studies were identified via PUBMED queries. Key search terms included:

- “Narcolepsy” and ‘‘cataplexy’’ in title/abstract (1435 articles);
- ‘‘Narcolepsy’’ and ‘‘emotions’’ in title/abstract (55 articles);
- “Narcolepsy” and “dream” in title/abstract (53 articles);
- “Narcolepsy” and “health” in title/abstract (197 articles).

We grouped the identified articles in the following categories:

1. Clinical aspects, diagnostic criteria, psychiatric comorbidity and life quality of narcolepsy;
2. Cataplexy: (a) clinical aspects, (b) role of the limbic system, (c) hypocretin deficit, (d) role of emotions, (e) emotional stimuli processing and coping strategies;
3. Dreaming in narcolepsy.

We focused on the role of emotionality in narcolepsy, excluding non-English articles. All the articles resulting from using this method and related to our focus were included. Following these criteria, we identified 64 publications which were estimated to be of interest for further examination.

## Physiopathology of cataplexy

It is well known that in NC patients the sudden drop in muscle tone is triggered by emotional factors such as laughing, joking, a pleasant surprise, and also anger [4–9]. This clinical feature suggests a close interaction between emotions and related anomalies in NC brain. For instance, neurophysiological evidence revealed a possible involvement of the amygdala in cataplexy. The amygdala is a limbic structure critically involved in emotional information processing in both animals and humans, as shown by neuroimaging, neurophysiological, and clinical studies (see [61–63]).

In the 1960s, the hypothesis of an involvement of this cerebral area in NC symptoms was proposed [64]. The first animal studies provided evidence for a relationship between alterations of amygdala and narcolepsy [e.g., 65, 66]. For instance, Siegel et al. [65] found elevated levels of axonal degeneration in the amygdala of narcoleptic dogs, which preceded or coincided with the disorder onset. This finding is coherent with the major symptoms of narcolepsy. An electrophysiological study on narcoleptic dogs [66] found a subpopulation of amygdala neurons associated with cataplexy, which significantly changed in activity before and during cataplectic attacks. This could have a role in mediation or modulation of these symptoms. Also, several human studies investigated the role of the amygdala in cataplexy. Hong et al. [67] used the Single Photon Emission Computed Tomography (SPECT) to localize cerebral perfusion differences during cataplexy. They found a hyperperfusion in the right amygdala during cataplectic attacks. However, this study included only two subjects. A recent functional magnetic resonance imaging (fMRI) study investigated the brain structures whose neural activity is specifically associated with emotion-induced cataplexy in 21 children/adolescent with NC [68]. Cataplectic attacks were elicited in 10 patients by using funny videos. The study showed an increase in blood oxygen level-depend signal in the amygdala during cataplectic attacks. Indirect evidence of amygdala involvement in NC also comes from psychophysiological research. Khatami et al. [69] investigated emotional process in NC and, in particular, emotional modulation of acoustic startle reflex (ASR) during the presentation of positive, neutral, and negative pictures selected from the International Affective Picture System (IAPS). The ASR is a kind of startle response triggered by threatening stimuli, and has been found to be dependent on limbic structures such as the amygdala in both animal and human studies [70–73]. The measurement of ASR allows assessing the role of the amygdala in NC. The results showed the absence in NC patients compared to controls of a startle potentiation to unpleasant stimuli, supporting the hypothesis of an amygdala dysfunction in this sleep disorder.

It should be considered that the amygdala is not the only limbic structure implicated in cataplexy. Indeed, several studies showed the involvement of the hypothalamus, considering it as the second main suprapontine brain site whose dysfunction might contribute to cataplexy. Joo et al. [74] found in a SPECT study a significant hypoperfusion in anterior hypothalamus in NC patients compared to controls. Also, an fMRI study [75] compared brain activity in NC patients and controls while they were exposed to humorous pictures, finding a reduction of hypothalamic response and enhancement of amygdala response in NC patients. These results suggested that cataplexy may depend on dysfunctional hypothalamic-amygdala interactions triggered by positive emotions. As mentioned above, narcolepsy has been found to be associated with a reduction or loss of the hypothalamic peptide hypocretin [76–82] that projects to the amygdala [83, 84]. The first evidence that hypocretin is involved in narcolepsy comes from animal models [22, 85, 86]. More interestingly, the link between hypocretin dysfunction and narcolepsy, and in particular NC, is also supported by human studies. Approximately 90% of patients with narcolepsy showed reduced levels of hypocretin in CSF [11], and *postmortem* studies confirmed the lack of detectable levels of this peptide in the cortex and the pons, in which normal hypocretinergic projections are found [78, 79].

Nevertheless, the dysfunction of the hypocretin system was observed only in NC, while a normal hypocretin concentration in CSF seemed to characterize the narcolepsy without cataplexy [11, 81, 87]. However, some cases of hypocretin deficiency without cataplexy have been described [14, 15]. Some animal studies suggested that the hypocretin neurons receive abundant projections from the limbic system [83, 84, 88], providing evidence that this neuronal system has close interactions with systems that regulate emotions. Besides, several studies supported the hypothesis that hypocretin is involved in a wide range of neurobehavioral processes, especially in situations of high motivational relevance [89]. Also, the hypocretin neurons activation was associated with acute stress [90], helping to organize stress responses, while chronic stress can disrupt hypocretin signaling in target regions [91]. Some studies found that the hypoactivity of the hypocretin system could yield depression-like symptoms and amotivational syndrome [90, 92–94]. Bearing in mind that NC is a chronic stress condition, the relationship between stress and hypocretin activation supports the hypothesis that neural input from the limbic system to the hypocretin neurons may be implicated in the pathophysiology of cataplexy. This link is also suggested by the role of emotional situations for cataplexy.

To sum up, neurophysiological and neurochemical findings indicate an implication of the limbic system in NC (e.g., [11, 67, 68, 74, 75]). Indeed, cataplexy seems to depend on dysfunctional hypothalamic-amygdala interactions associated with an alteration of the hypocretin levels. Hence, the involvement of emotional brain network in the physiopathology of cataplexy could explain why emotional inputs triggered cataplexy.

## Cataplexy and emotions

Lazarus [95] stated that emotions represent the “wisdom of the ages”, providing time-tested responses to adaptive problems. Emotions arise when something important to us is at stake, but they do not force us to respond in specific ways, they only make certain responses more probable. This malleability allows us emotional regulation, meaning processes that influence how we experience and express emotions [96]. This focus is particularly interesting for narcolepsy. Considering the crucial role of emotions in cataplexy [4–9], it could be hypothesized a relationship between emotional reactions and cataplexy attacks, mediated by coping strategies. In particular, coping strategies could consist of an emotional constriction that serves as an adaptive response to ward off cataplexy [97, 98]. The strategy to keep down expression of their own emotions could reduce or suppress emotion-related behaviors (e.g., laughing, crying) that usual trigger cataplexy [97, 98]. This hypothesis is also supported by the relationship between disease duration and cognitive responses to the emotional stimuli: a longer disease seems to be associated with cognitive suppression [98]. Consistently, a prospective study on children showed for the first time a reduction of cataplexy severity after about 3 years after the NC onset [99].

With regards to cataplexy and coping strategies, some studies focused on the emotional reactions in NC patients using standard stimuli to elicit emotions and investigates the emotional stimuli processing in these subjects. Tucci et al. [97] investigated the emotional visual stimuli processing in NC patients, using the IAPS [100–102]. Muscular, autonomic, and cognitive reactions were measured while the subjects were exposed to pictures with positive, neutral and negative valence. They found that these reactions were attenuated in NC patients compared to controls. In particular, they showed the lowest responses with negative pictures. Also, De Zambotti et al. [98] used the IAPS to investigate hemodynamic and behavioral responses during emotional visual stimulation in NC patients. The hemodynamic responses to pictures were the same for NC patients and controls, while differences were found for behavioral responses: NC patients reported lower arousal scores associated to positive and neutral stimuli, a status of less pleasantness induced by pleasant stimulation, lower valence scores associated to pleasant stimuli and lower score at the “focus on and venting of emotions” dimensions of coping. Another study investigated facial emotional expressions (fear, happiness and sadness) and emotional regulation strategies using a self-rated questionnaire in NC compared to central hypersomnia without cataplexy and healthy controls. No differences have been found among the groups, namely the NC showed a typical emotional judgment ability and emotional regulation strategies, contrary to the authors’ initial hypothesis [103] These findings are counterintuitive considering that several studies found that amygdala damage is related to impairment in recognition of emotional facial expressions [104]. Nevertheless, metabolic changes of the amygdala in NC patients may be localized in only one hemisphere (i.e., the right hemisphere), being without any consequence on facial expression recognition [105]. Hence, the authors suggested that normal functioning of the amygdala may be unnecessary to normal performance on this tasks and narcoleptic patients may recruit different networks to produce a normal recognition performance [103].

Other studies investigated the humor stimuli processing in NC, considering that the most frequent emotional trigger for the cataplexy seems to be the laughter [e.g., 8, 9]. For example, Susta et al. [106] used a protocol using audio recordings aimed to trigger laughter in NC patients and controls. They registered cortical brain activity by quantitative electroencephalography (qEEG) source localization to calculate the sequence of brain areas involved in laughter processing. The results showed a different pre-laughter activation between NC patients and controls. The gyrus orbitalis, the gyrus rectus and the gyrus occipitalis inferior were activated in controls, and the gyrus paracentralis, the gyrus cingularis, and the cuneus were activated in the patients.

Some authors measured the H-reflex during emotive stimuli, which relies on contraction of the flexors of the calf because of the excitation in the tibial nerve. The reflex was reduced during laughter in healthy subjects, giving the feeling of lack of strength [107]. It should be noted that this effect may have a role in cataplexy. The H-reflex is the only neurophysiological variable which is altered in this phenomenon, disappearing cataplectic attacks [108–110]. Another study investigated the neurophysiological effects of startle and laughter in NC [111]. They found a decrement of H-reflex during laughter, without differences between NC and controls and with increased startle response in NC [111].

Most of these studies clearly showed how reactions to emotional stimuli are different in NC patients compared to controls [87, 97, 98, 106]. Some works found emotional reactions significantly different between NC patients and controls in response to unpleasant situations [97, 111], while others in response to pleasant situations [98, 106]. The heterogeneous results among pleasant and unpleasant stimuli may depend on methodological differences, like a different input use (e.g., visual, audio) or output measure (e.g., muscular, autonomic, cognitive, hemodynamic and behavioral responses, qEEG, H-reflex). However, all these findings suggest an impairment in emotions processing in NC, raising the possibility that these alterations are part of compensatory strategies to avoid or reduce in frequency cataplexy attacks [97–99]. However, this hypothesis does not rule out the possibility that the impairment in emotions processing in NC could be in relation with the impairment of emotional brain network (e.g., [11, 67, 68, 74, 75]).

According to the appraisal theories, emotions depend on the personal evaluation of the events [112]. It has been demonstrated that appraisal can determine different emotional reactions and modulate their intensity [113]. In other words, personal evaluation of situations as less pleasant or unpleasant could be the result of a cognitive approach to minimize the impact of emotion, for instance through the suppression which is the attempt to decrease or inhibit emotion-expressive behavior [114]. Therefore, it could be hypothesized the same cognitive mechanism in NC patients who develop unware adaptive strategies to face emotions and, specifically, to avoid cataplectic attacks. These hypotheses on coping strategies in NC do not explain the increasing startle response in NC found by Lammers et al. [111]. According to the authors [111], these findings may depend on the brain or neurochemical anomalies that characterized NC. For instance, axonal degeneration in the amygdala and the medial septum region was found in narcoleptic dogs [65], and both these areas are known to be involved in startle responses. Furthermore, an increased startle response could be explained by an altered adrenergic tone, that characterized both startle reflexes [115, 116] and cataplexy [117]. So Lammers et al. [111] speculated that the exaggerated startle response could be a side effect, an increased sensitivity for adrenergic stimulation as partial compensation of their propensity for cataplexy.

Finally, it should be considered that all the studies mentioned have the limit of not evaluating cataplexy, but its apparent subclinical expressions. Namely, no cataplectic attack was elicited during the experiments. This may be explained by the use of stimuli with lower emotional impact than real-world stimuli. Despite these subclinical data, different patterns of emotional management and expression have been reported in NC compared to controls, which are probably linked to specific styles of coping.

## Mental sleep activity in narcolepsy and emotional experience

Some studies showed that emotional features of dreams are related to the activation of the limbic system [118–120]. However, it should be underlined that dreaming is a peculiar object of study because of the methodological limitation to a reliable access to the mental sleep activity (MSA), namely the products of mental processing during sleep which are reported upon awakening in the form of dream report [121–123]. Microstructural analyses also found a link between emotional features in dreams and the limbic system, investigating the relationships between volumetric and ultrastructural measures of the hippocampus-amygdala complex and specific qualitative features of dreaming [124]. In particular, bizarreness of dream reports was negatively correlated with the left amygdala volume and positively correlated with the microstructural integrity (i.e., lower mean diffusivity) of the right amygdala, while emotional load was directly proportional to the microstructural integrity (i.e., lower mean diffusivity) of the left amygdala; on the other hand, the volume of the right hippocampus was negatively associated with bizarreness [124]. In other words, dream contents characterized by higher level of bizarreness were related with smaller left amygdala, smaller right hippocampus and lower mean diffusivity of the right amygdala, while dreams characterized by high emotional load were related to the low mean diffusivity of the left amygdala. This finding was subsequently confirmed in another study on patients with Parkinson’s Disease (PD), showing for the first time in this population that the dopamine network plays a key role in dream experience. A higher dopamine agonist dosage was associated with qualitatively impoverished MSA, as expressed by lower bizarreness and emotional load values [125]. Alterations in dopaminergic levels also characterize narcolepsy, and indeed Modafinil is used to enhance vigilance in treatment of this disease [126, 127] because of its ability to increase cerebral levels of dopamine [128, 129]. Keeping in mind the role of dopamine network in dreaming [125] and its impairment in narcolepsy [126, 127], it is possible to speculate that dream experience in this sleep disorder could be affected by neurochemical unbalance.

Considering all these findings [124, 125], the crucial role of emotions in narcolepsy [4, 6–9, 69, 97, 98, 106, 111] and an impairment of the limbic system in these subjects (e.g., [11, 67, 68, 74, 75]), the investigation on emotional experience during MSA for this sleep disorder deserves interest. Narcoleptic patients often report an abundant production of vivid, bizarre and frightening MSA [130]. A high prevalence of aggressive dreams was found, including dreams with aggressive sexual themes [131]. Furthermore, some studies revealed that narcoleptic patients, with or without cataplexy, have more negatively toned and bizarre dreams and significantly more terrifying and repetitive dreams in narcoleptic patients compared to insomnia patients [132]. However, it should be noted that some authors also found positive emotions in narcoleptic dreams [133]. Other authors [134] posited that NC exaggerates the emotional aspects of REM dreams, probably because of the alteration of neurobiological systems that support cognitive-emotional functions. They investigated the emotional experience during REM sleep in these patients and observed more intense emotions –especially- anxiety/fear and following joy/elation [134] and more bizarre and vivid contents [135].

Regarding the awareness of dreaming experience in these patients, a recent study investigated the prevalence of dream-reality confusion in NC. Dream delusions are extremely common in this disorder, and patients with NC were much more likely to report mistaking dream experiences as true memories, in comparison with healthy controls [136]. Conversely, other results suggested that these patients were more often aware of their state of consciousness during dreaming than healthy subjects (i.e., they knew that the dream experience was not real) [133]. Furthermore, it was found a higher prevalence of lucid dreaming in NC and N patients, compared to healthy subjects [137, 138]. Lucid dreaming is the experience of being aware of dreaming while asleep and continuing to dream [139], and this awareness could be used to modify unpleasant narcoleptic dreams. Meaidi et al. [140] found a significant increase in lucid dreaming of N and NC compared to healthy controls, without any difference in emotional contents in the frequency of nightmares. Differently, another study showed an increase of nightmares for these patients [141]. Pisko et al. [141] showed that the prevalence of vivid but not unpleasant dreams for N and NC patients was 26%, with a higher frequency for NC patients. This study also found that the prevalence of nightmares was 33%, without difference between N and NC, while the prevalence of nightmares in general population was around 5% [142, 143]. According to Meaidi et al. [140], the intensification of lucid dreaming in N and NC patients compared to healthy controls could explain the low incidence of nightmares in the clinical group, suggesting that lucid dream may be part of a coping strategy and may be useful to treat nightmare disorders. For this reason, the so-called “lucid dreams” have been repeatedly proposed as an effective therapy for nightmare disorder [144–146]. A strategy to induce lucid dreaming in healthy subjects is the wake-up-back-to-bed technique, in which subjects are awakened in the early morning hours and go back to sleep after a period of wakefulness [147, 148]. Perhaps, same narcoleptic sleep peculiarities (i.e., fragmented night sleep, SOREMs) might have similar effects to the wake-up-back-to-bed technique in healthy, promoting cortical arousal that facilitated lucid dreaming.

It should be noted that narcoleptic patients, even during sleep, have an emotional experience different from healthy subjects [113, 131, 134, 135, 141]. Interestingly, N and NC could use coping strategies also during their sleep, avoiding unpleasant MSA through lucid dreaming [138, 140]. Moreover, this kind of emotional experience during sleep, i.e., so vivid, bizarre and frightening [113, 131, 132, 134, 135, 141], could be partly linked to an emotional experience in waking state, like a possible consequence of emotional inhibition and defensive attitude during the daytime. Finally, emotional experience during MSA seems not influenced by the physiopathology of cataplexy, since there is no difference between N and NC [137, 138, 140, 141].

## Future perspectives

Overall, the discussed results emphasize that the relationship between NC and emotional processes is still poorly understood. On this basis, some further considerations and insights for future studies seem appropriate.

Firstly, considering the hypothesis about the coping strategies of patients with NC, further investigations are necessary to understand which strategy should be applied and to what extent this could represent an adaptive behavior by modulating the occurrence of symptoms. In this vein, the ability to express emotions may affect cataplexy, and the presence/absence of alexithymia in NC should be assessed. A study reported a higher score on Toronto Alexithymia Scale-20 (TAS-20) [149] in a group of narcoleptic patients than in a control group [58]. These patients could actively control the expression of their emotions, avoiding a contact with their emotions to prevent some unpleasant symptoms, like cataplexy attacks for NC. This viewpoint becomes promising especially considering that NC is a chronic disorder and it could be associated with a secondary form of alexithymia, as an attempt to cope with the stress of situation (e.g., [150–153]). In other words, secondary alexithymia could be considered as a consequence of the illness-related stress, like a defense mechanism against highly emotional events [153, 154].

Moreover, subjects with narcolepsy can experience a gradual reduction of quality of life and exposed to an increased risk of work-related and transit accidents, sexual dysfunctions and neuropsychological alterations [155, 156], developing dysfunctional coping strategies. For these reasons, it could be expected that alexithymia levels change during NC progression. In this vein, alexithymia levels should be assessed in different phases of the disease, by longitudinal designs aimed to investigate if and how emotional awareness and expression change as symptoms progress.

Moreover, alexithymia may be related to dream alterations in NC [113, 131, 134, 135, 141]. Although not completely consistent, some independent findings on alexithymia compared to healthy subjects suggested that dream recall rate was lower and more impoverished [58, 157–165], while nightmares were more frequent or distressing [58, 164, 166]. Since the relationship between alexithymia and dreaming involves processes regulating emotion during both wakefulness and dreaming, this issue should be further addressed.

Furthermore, we have underlined that subjects with NC report qualitatively rich dream contents [113, 131, 135, 141, 166], suggesting that emotional memory consolidation in these patients is not compromised. In this respect, we have to consider that alteration of the limbic network in these patients (e.g., [20, 67, 68, 74, 75]) and the findings on the neural substrates of dreaming [124, 125] are not always consistent. For instance, the emotional load characterizing the core symptoms of PTSD-such as nightmares-seems to be related to the hyperactivation of the amygdala [167, 168]. Also, REM sleep could amplify the altered function of the amygdala [168]. Several functional neuroimaging human studies revealed that emotional network, including the amygdala, was activated during REM sleep [118, 169–172], even if these studies still were based on the equation “REM sleep = dreaming”.

Moreover, a stereo-EEG study on pharmaco-resistant epileptic patients found a transient activation of the amygdala which was time-locked to the onset of REM sleep, suggesting that this activation may be involved in adding an emotional tone to dream contents [173]. Also, recent investigations confirmed the relationship between some structural parameters of the amygdala and the hippocampus and the emotional load of dream reports [124, 125]. Taken together, these findings support the hypothesis that the activation of the limbic system may play a crucial role in the dreaming, but how the impairment of the limbic system affects MSA in NC is still unknown. In our opinion, this issue requires further investigations. Furthermore, studies on emotional experience during MSA did not find differences between N and NC [137, 138, 140, 141], suggesting that this is not influenced by the physiopathology of cataplexy. Hence, studies that investigated in N and NC the specific role of hypocretin during MSA also are needed.

Finally, it should be mentioned that EEG studies provided some indications about the relationship between brain networks and emotional memories. In particular, the prefrontal theta activity during REM sleep has been related to emotional memory consolidation [174]. The same EEG activity (5–7 Hz) during REM sleep predicted the subsequent dream recall, suggesting shared mechanisms in the retrieval of episodic memory across different states of consciousness [175, 176]. However, in NC just a few studies have been carried out to identify EEG correlates of dreaming. To the best of our knowledge, the only study in this direction found a relationship between the gamma EEG activity and lucid dreams, and also a reduced delta activity seems related to the lucidity of dreams [138]. This result is not coherent with the studies mentioned above [175, 176], while supports the view of a direct relation between dream recall rate and decreased cortical activation [177, 178]. According to these considerations and to the fact that NC patients show higher dream recall rate than healthy population [137, 138, 179], we suggest that other studies should be developed to investigate the EEG correlates of dream recall in this population, examining -specifically- the relation between EEG pattern and emotional dream contents.

## Concluding remarks

This review summarizes the studies on the relationship between emotional experiences and narcolepsy. Neurophysiological and neurochemical findings support the hypothesis of the involvement of the limbic system in the physiopathology of cataplexy [e.g., 20, 67, 68, 74, 75], and this could explain the relationship between cataplexy and emotional inputs. Furthermore, behavioral studies suggest an impairment in emotions processing in NC, like a possible coping strategy to avoid or reduce in frequency cataplexy attacks [97, 98, 106] and, consistently, these patients could use these coping strategies even during sleep, avoiding unpleasant MSA through lucid dreaming [138, 140]. Moreover, about dreaming in NC, we have pointed out that these patients report peculiar dream contents, characterized by more vivid, bizarre and frightening contents than those of healthy subjects [113, 131, 134, 135, 141]. Therefore, emotional memory consolidation seems not being altered, although several studies suggest the impairment of the limbic system in this disease (e.g., [20, 67, 68, 74, 75]).

## References

[1] Vogel GW (1960). Studies in the psychophysiology of dreams III. The dream of narcolepsy. Arch Gen Psychiatry..

[2] Dement WC, Rechtschaffen A, Gulevich G (1966). The nature of the narcoleptic sleep attack. Neurology.

[3] American Academy of Sleep Medicine (2014). International classification of sleep disorders.

[4] Dauvilliers Y, Arnulf I, Mignot E (2007). Narcolepsy with cataplexy. Lancet.

[5] Overeem S, Mignot E, van Dijk JG, Lammers GJ (2001). Narcolepsy: clinical features, new pathophysiologic insights, and future perspectives. J Clin Neurophysiol..

[6] Thorpy MJ (2006). Cataplexy associated with narcolepsy: epidemiology, pathophysiology, and management. CNS Drugs..

[7] Scammell TE (2003). The neurobiology, diagnosis, and treatment of narcolepsy. Ann Neurol..

[8] Anic-Labat S, Guilleminault C, Kraemer HC, Meehan J, Arrigoni J, Mignot E (1999). Validation of a cataplexy questionnaire in 983 sleep-disorders patients. Sleep..

[9] Krahn LE, Lymp JF, Moore WR, Slocumb N, Silber MH (2005). Characterizing the emotions that trigger cataplexy. J Neuropsychiatry Clin Neurosci..

[10] American Academy of Sleep Medicine, eds. International Classification of Sleep Disorders. 2nd ed.: Diagnostic and Coding Manual. American Academy of Sleep Medicine: Westchester; 2005.

[11] Mignot E, Lammers GJ, Ripley B, Okun M, Nevsimalova S, Overeem S, Vankova J, Black J, Harsh J, Bassetti C, Schrader H, Nishino S (2002). The role of cerebrospinal fluid hypocretin measurement in the diagnosis of narcolepsy and other hypersomnias. Arch Neurol..

[12] Nishino S, Ripley B, Overeem S, Lammers GJ, Mignot E (2000). Hypocretin (orexin) deficiency in human narcolepsy. Lancet.

[13] Kanbayashi T, Inoue Y, Chiba S, Aizawa R, Saito Y, Tsukamoto H, Fujii Y, Nishino S, Shimizu T (2002). CSF hypocretin-1 (orexin-A) concentrations in narcolepsy with and without cataplexy and idiopathic hypersomnia. J Sleep Res.

[14] Ripley B, Overeem S, Fujiki N, Nevsimalova S, Uchino M, Yesavage J, Di Monte D, Dohi K, Melberg A, Lammers GJ, Nishida Y, Roelandse FW, Hungs M, Mignot E, Nishino S (2001). CSF hypocretin/orexin levels in narcolepsy and other neurological conditions. Neurology..

[15] Andlauer O, Moore H, Hong SC, Dauvilliers Y, Kanbayashi T, Nishino S, Han F, Silber MH, Rico T, Einen M, Kornum BR, Jennum P, Knudsen S, Nevsimalova S, Poli F, Plazzi G, Mignot E (2012). Predictors of hypocretin (orexin) deficiency in narcolepsy without cataplexy. Sleep..

[16] Westphal C (1877). Eigentümliche mit Einschlafen verbundene Anfälle. Arch f Psych..

[17] Gelineau JBE (1880). De la Narcolepsy (I). Gaz des Hôp.

[18] Gelineau JBE (1880). De la Narcolepsy (II). Gaz des Hôp.

[19] Pagel JF, Goswami M, Thorpy M, Pandi-Perumal S (2016). Psychoanalysis and Narcolepsy. Narcolepsy.

[20] Freud S (1900). Die Traumdeutung.

[21] Langworthy OR, Betz BJ (1944). Narcolepsy as a type of response to emotional conflicts. Psychosom Med..

[22] Switzer RE, Berman AD (1956). Comments and observations on the nature of narcolepsy. Ann Intern Med..

[23] Morgenstern AL (1965). The neurotic component of narcolepsy. Am J Psychiatry.

[24] Lin L, Faraco J, Li R, Kadotani H, Rogers W, Lin X, Qiu X, de Jong PJ, Nishino S, Mignot E (1999). The sleep disorder canine narcolepsy is caused by a mutation in the hypocretin (orexin) receptor 2 gene. Cell.

[25] Chemelli RM, Willie JT, Sinton CM, Elmquist JK, Scammell T, Lee C, Richardson JA, Williams SC, Xiong Y, Kisanuki Y, Fitch TE, Nakazato M, Hammer RE, Saper CB, Yanagisawa M (1999). Narcolepsy in orexin knockout mice: molecular genetics of sleep regulation. Cell.

[26] Hara J, Beuckmann CT, Nambu T, Willie JT, Chemelli RM, Sinton CM, Sugiyama F, Yagami K, Goto K, Yanagisawa M, Sakurai T (2001). Genetic ablation of orexin neurons in mice results in narcolepsy, hypophagia, and obesity. Neuron.

[27] Lishman A (1998). Organic psychiatry.

[28] Morse AM, Sanjeev K (2018). Narcolepsy and psychiatric disorders: comorbidities or shared pathophysiology?. Med Sci..

[29] Daniels E, King MA, Smith IE, Shneerson JM (2001). Health-related quality of life in narcolepsy. J. Sleep Res..

[30] Dauvilliers Y, Paquereau J, Bastuji H, Drouot X, Weil JS, Viot-Blanc V (2009). Psychological health in central hypersomnias: the french harmony study. J Neurol Neurosurg Psychiatr..

[31] Fortuyn HAD, Mulders P, Renier W, Buitelaar J, Overeem S (2011). Narcolepsy and psychiatry: an evolving association of increasing interest. Sleep Med.

[32] Fortuyn HAD, Lappenschaar G, Furer JW, Hodiamont PP, Rijnders CA, Renier WO, Buitelaar JK, Overeem S (2010). Anxiety and mood disorders in narcolepsy. Gen Hosp Psychiatr..

[33] Vourdas A, Shneerson J, Gregory C, Smith IE, King MA, Morrish E, McKenna PJ (2002). Narcolepsy and psychopathology: is there an association?. Sleep Med.

[34] Brundin L, Björkqvist M, Petersén Å, Träskman-Bendz L (2007). Reduced orexin levels in the cerebrospinal fluid of suicidal patients with major depressive disorder. Eur Neuropsychopharmacol.

[35] Schmidt FM, Arendt E, Steinmetzer A, Bruegel M, Kratzsch J, Strauss M, Baum P, Hegerl U, Schönknecht P (2011). CSF-hypocretin-1 levels in patients with major depressive disorder compared to healthy controls. Psychiatr Res..

[36] Canellas F, Lin L, Julià MR, Clemente A, Vives-Bauza C, Ollilla HM, Chul Hong S, Arboleya SM, Einen MA, Faraco J (2014). Dual cases of type 1 narcolepsy with schizophrenia and other psychotic disorders. J Clin Sleep Med.

[37] Fortuyn HAD, Lappenschaar G, Nienhuis FJ, Furer JW, Hodiamont PP, Rijnders CA, Lammers GJ, Renier WO, Buitelaar JK, Overeem S (2009). Psychotic symptoms in narcolepsy: phenomenology and a comparison with schizophrenia. Gen Hosp Psychiatr..

[38] Taylor SF, Tandon R, Shipley JE, Eiser AS, Goodson J (1991). Sleep onset REM periods in schizophrenic patients. Biol Psychiatr..

[39] D’Agostino A, Limosani I, Goswami M, Thorpy M, Pandi-Perumal S (2016). Hypnagogic hallucinations and sleep paralysis. Narcolepsy.

[40] Douglass AB, Hays P, Pazderka F, Russell JM (1991). Florid refractory schizophrenias that turn out to be treatable variants of HLA-associated narcolepsy. J Nerv Ment Dis.

[41] Bhat SK, Galang R (2002). Narcolepsy presenting as schizophrenia. Am J Psychiatry.

[42] Szűcs A, Janszky J, Hollo A (2003). Misleading hallucinations in unrecognized narcolepsy. Acta Psychiatr Scand.

[43] Talih FR (2011). Narcolepsy presenting as schizophrenia. A literature review and two case reports. Innov Clin Neurosci..

[44] Jardri R, Bartels-Velthuis AA, Debbané M, Jenner JA, Kelleher I, Dauvilliers Y, Plazzi G, Demeulemeester M, David CN, Rapoport J, Dobbelaere D, Escher S, Fernyhough C (2014). From phenomenology to neurophysiological understanding of hallucinations in children and adolescents. Schizophr Bull..

[45] Sansa G, Gavaldà A, Gaig C, Monreal J, Ercilla G, Casamitjana R, Ribera G, Iranzo A, Santamaria J (2016). Exploring the presence of narcolepsy in patients with schizophrenia. BMC Psychiatry..

[46] Dauvilliers Y, Gaig C, Barateau L, Graus F, Iranzo A, Lopez R, Santamaria J (2016). Absence of NMDA receptor antibodies in the rare association between type 1 narcolepsy and psychosis. Sci Rep..

[47] Plazzi G, Fabbri C, Pizza F, Serretti A (2015). Schizophrenia-Like Symptoms in Narcolepsy Type 1: shared and Distinctive Clinical Characteristics. Neuropsychobiology.

[48] Tempesta D, Socci V, De Gennaro L, Ferrara M. Sleep and emotional processing. Sleep Med Rev. 2017, pii: S1087-0792(17)30153-3. 10.1016/j.smrv.2017.12.005 29395984

[49] Horne JA (1985). Sleep function, with particular reference to sleep deprivation. Ann Clin Res..

[50] Dinges DF, Pack F, Williams K, Gillen KA, Powell JW, Ott GE, Aptowicz C, Pack AI (1997). Cumulative sleepiness, mood disturbance, and psychomotor vigilance performance decrements during a week of sleep restricted to 4-5 hours per night. Sleep.

[51] Zohar D, Tzischinsky O, Epstein R, Lavie P (2005). The effects of sleep loss on medical residents’ emotional reactions to work events: a cognitive-energy model. Sleep.

[52] Kahn-Greene ET, Killgore DB, Kamimori GH, Balkin TJ, Killgore WD (2007). The effects of sleep deprivation on symptoms of psychopathology in healthy adults. Sleep Med..

[53] McCrae CS, McNamara JP, Rowe MA, Dzierzewski JM, Dirk J, Marsiske M, Craggs JG (2008). Sleep and affect in older adults: using multilevel modeling to examine daily associations. J Sleep Res..

[54] Norlander T, Johansson A, Bood A (2005). The affective personality: its relation to quality of sleep, well-being and stress. Soc Behav Personal Int J..

[55] Scott BA, Judge TA (2006). Insomnia, emotions and job satisfaction: a multilevel study. J Manag..

[56] Buysse DJ, Thompson W, Scott J, Franzen PL, Germain A, Hall M (2007). Daytime symptoms in primary insomnia: a prospective analysis using ecological momentary assessment. Sleep Med..

[57] Klůzová Kráčmarová L, Plháková A (2015). Nightmares and their consequences in relation to state factors, absorption, and boundaries. Dreaming.

[58] Nielsen T, Levrier K, Montplaisir J (2011). Dreaming correlates of alexithymia among sleep-disordered patients. Dreaming.

[59] Crisp AH, Matthews BM, Oakey M, Crutchfield M (1990). Sleepwalking, night terrors, and consciousness. BMJ..

[60] American Psychiatric Association (2013). Diagnostic and Statistical Manual of Mental Disorders.

[61] LeDoux JE (2000). Emotion circuits in the brain. Annu Rev Neurosci.

[62] Zald DH (2003). The human amygdala and the emotional evaluation of sensory stimuli. Brain Res Brain Res Rev.

[63] Vuilleumier P (2005). How brains beware: neural mechanisms of emotional attention. Trends Cogn Sci.

[64] Vizioli R (1964). Neurophysiological bases of catalepsy. Electroencephalogr Clin Neurophysiol.

[65] Siegel JM, Nienhuis R, Gulyani S (1999). Neuronal degeneration in canine narcolepsy. J Neurosci.

[66] Gulyani S, Wu MF, Nienhuis R, John J, Siegel JM (2002). Cataplexy-related neurons in the amygdala of the narcoleptic dog. Neuroscience.

[67] Hong SB, Tae WS, Joo EY (2006). Cerebral perfusion changes during cataplexy in narcolepsy patients. Neurology..

[68] Meletti S, Vaudano AE, Pizza F (2015). The brain correlates of laugh and cataplexy in childhood narcolepsy. J Neurosci.

[69] Khatami R, Birkmann S, Bassetti C (2007). Amygdala dysfunction in narcolepsy-cataplexy. J Sleep Res.

[70] Davis M (1997). Neurobiology of fear responses: the role of the amygdala. J Neuropsychiatr Clin Neurosci..

[71] Grillon C, Baas J (2003). A review of the modulation of the startle reflex by affective states and its application in psychiatry. Clin Neurophysiol.

[72] Hitchcock JM, Sananes CB, Davis M (1989). Sensitization of the startle reflex by footshock: blockade by lesions of the central nucleus of the amygdala or its efferent pathway to the brainstem. Behav Neurosci.

[73] Pissiota A, Frans O, Michelgard A, Appel L, Langstrom B, Flaten MA, Fredrikson M (2003). Amygdala and anterior cingulate cortex activation during affective startle modulation: a PET study of fear. Eur J Neurosci.

[74] Joo E, Hong SB, Tae WS, Kim JH, Han SJ, Cho YW (2005). Cerebral perfusion abnormality in narcolepsy with cataplexy. Neuroimage.

[75] Schwartz S, Ponz A, Poryazova R, Werth E, Boesiger P, Khatami R (2008). Abnormal activity in hypothalamus and amygdala during humour processing in human narcolepsy with cataplexy. Brain.

[76] de Lecea L, Kilduff TS, Peyron C, Gao X, Foye PE, Danielson PE, Fukuhara C, Battenberg EL, Gautvik VT, Bartlett FS, Frankel WN, van den Pol AN, Bloom FE, Gautvik KM, Sutcliffe JG (1998). The hypocretins: hypothalamus-specific peptides with neuroexcitatory activity. Proc Natl Acad Sci USA.

[77] Sakurai T, Amemiya A, Ishii M, Matsuzaki I, Chemelli RM, Tanaka H, Williams SC, Richardson JA, Kozlowski GP, Wilson S, Arch JR, Buckingham RE, Haynes AC, Carr SA, Annan RS, McNulty DE, Liu WS, Terrett JA, Elshourbagy NA, Bergsma DJ, Yanagisawa M (1998). Orexins and orexin receptors: a family of hypothalamic neuropeptides and G protein-coupled receptors that regulate feeding behavior. Cell.

[78] Peyron C, Faraco J, Rogers W, Ripley B, Overeem S (2000). A mutation in a case of early onset narcolepsy and a generalized absence of hypocretin peptides in human narcoleptic brains. Nat Med.

[79] Thannickal TC, Moore RY, Nienhuis R, Ramanathan L, Gulyani S (2000). Reduced number of hypocretin neurons in human narcolepsy. Neuron.

[80] Thannickal TC, Siegel JM, Nienhuis R, Moore RY (2003). Pattern of hypocretin (orexin) soma and axon loss, and gliosis, in human narcolepsy. Brain Pathol.

[81] Nishino S, Ripley B, Overeem S, Lammers GJ, Mignot E (2000). Hypocretin (orexin) deficiency in human narcolepsy. Lancet.

[82] Nishino S, Fujiki N, Ripley B, Sakurai E, Kato M (2001). Decreased brain histamine content in hypocretin/orexin receptor-2 mutated narcoleptic dogs. Neurosci Lett.

[83] Sakurai T, Nagata R, Yamanaka A, Kawamura H, Tsujino N, Muraki Y, Kageyama H, Kunita S, Takahashi S, Goto K, Koyama Y, Shioda S, Yanagisawa M (2005). Input of orexin/hypocretin neurons revealed by a genetically encoded tracer in mice. Neuron.

[84] Yoshida K, McCormack S, España RA, Crocker A, Scammell TE (2006). Afferents to the orexin neurons of the rat brain. J Comp Neurol..

[85] Chemelli RM, Willie JT, Sinton CM, Elmquist JK, Scammell T, Lee C, Richardson JA, Williams SC, Xiong Y, Kisanuki Y, Fitch TE, Nakazato M, Hammer RE, Saper CB, Yanagisawa M (1999). Narcolepsy in orexin knockout mice: molecular genetics of sleep regulation. Cell.

[86] Hara J, Beuckmann CT, Nambu T, Willie JT, Chemelli RM, Sinton CM, Sugiyama F, Yagami K, Goto K, Yanagisawa M, Sakurai T (2001). Genetic ablation of orexin neurons in mice results in narcolepsy, hypophagia, and obesity. Neuron.

[87] Kanbayashi T, Inoue Y, Chiba S, Aizawa R, Saito Y, Tsukamoto H, Fujii Y, Nishino S, Shimizu T (2002). CSF hypocretin-1 (orexin-A) concentrations in narcolepsy with and without cataplexy and idiopathic hypersomnia. J Sleep Res.

[88] Winsky-Sommerer R, Yamanaka A, Diano S, Borok E, Roberts AJ, Sakurai T, Kilduff TS, Horvath TL, de Lecea L (2004). Interaction between the corticotropin-releasing factor system and hypocretins (orexins): a novel circuit mediating stress response. J Neurosci.

[89] Mahler SV, Moorman DE, Smith RJ, James MH, Aston-Jones G (2014). Motivational activation: a unifying hypothesis of orexin/hypocretin function. Nat Neurosci.

[90] Yeoh JW, Campbell EJ, James MH, Graham BA, Dayas CV (2014). Orexin antagonists for neuropsychiatric disease: progress and potential pitfalls. Front. Neurosci..

[91] Liu RJ, Aghajanian GK (2008). Stress blunts serotonin- and hypocretin-evoked EPSCs in prefrontal cortex: role of corticosterone-mediated apical dendritic atrophy. Proc Natl Acad Sci USA.

[92] Johnson PL, Molosh A, Fitz SD, Truitt WA, Shekhar A (2012). Orexin, stress and anxiety/panic states. Prog Brain Res.

[93] Salomon RM (2003). Diurnal variation of cerebrospinal fluid hypocretin-1 (Orexin-A) levels in control and depressed subjects. Biol Psychiatry.

[94] Bayard S, Dauvilliers YA (2013). Reward-based behaviors and emotional processing in human with narcolepsy-cataplexy. Front Behav Neurosci..

[95] Lazarus RS (1991). Progress on a cognitive-motivational-relational theory of emotion. Am Psychol.

[96] Gross JJ (1998). The emerging field of emotion regulation: an integrative review. Rev Gen Psychol.

[97] Tucci V, Stegagno L, Vandi S, Ferrillo F, Palomba D, Vignatelli L, Ferini-Strambi L, Montagna P, Plazzi G (2003). Emotional information processing in patients with narcolepsy: a psychophysiologic investigation. Sleep.

[98] de Zambotti M, Pizza F, Covassin N, Vandi S, Cellini N, Stegagno L, Plazzi G (2014). Facing emotions in narcolepsy with cataplexy: haemodynamic and behavioural responses during emotional stimulation. J Sleep Res..

[99] Pizza F, Franceschini C, Peltola H (2013). Clinical and polysomnographic course of childhood narcolepsy with cataplexy. Brain.

[100] Lang PJ, Greenwald MK. The International Affective Picture System standardization procedure and initial group results for affective judgments: technical report IB. Gainesville, FL: 1988, The Center for Research in Psychophysiology, University of Florida.

[101] Lang PJ, Ohman A, Vaitl D (1988). The international affective picture system [Photographic slides].

[102] Lang PJ, Bradley MM, Cuthbert BN (1999). International affective picture system (IAPS): technical manual and affective ratings.

[103] Bayard S, Langenier MC, Dauvilliers Y (2013). Facial expression recognition and emotional regulation in narcolepsy with cataplexy. J Sleep Res.

[104] Adolphs R, Tranel D, Damasio H, Damasio A (1994). Impaired recognition of emotion in facial expressions following bilateral damage to the human amygdala. Nature.

[105] Poryazova R, Schnepf B, Werth E (2009). Evidence for metabolic hypothalamo-amygdala dysfunction in narcolepsy. Sleep.

[106] Susta M, Nemcova V, Bizik G, Sonka K (2017). Emotion stimulus processing in narcolepsy with cataplexy. J Sleep Res..

[107] Overeem S, Lammers GJ, van Dijk JG (1999). Weak with laughter. Lancet.

[108] Guilleminault C, Guilleminault C, Dement WC, Passouant P (1976). Cataplexy. Narcolepsy.

[109] Guilleminault C, Gelb M, Fahn S, Hallett M, Luders HO, Marsden CD (1995). Clinical aspects and features of cataplexy. Negative motor phenomena.

[110] Hishikawa Y, Shimizu T, Fahn S, Hallett M, Luders HO, Marsden CD (1995). Physiology of REM sleep, cataplexy, and sleep paralysis. Negative motor phenomena.

[111] Lammers GJ, Overeem S, Tijssen MA, van Dijk JG (2000). Effects of startle and laughter in cataplectic subjects: a neurophysiological study between attacks. Clin Neurophysiol..

[112] Smith C, Ellsworth P (1987). Patterns of appraisal and emotion related to taking an exam. J Pers Soc Psychol.

[113] Siemer M, Mauss I, Gross J (2007). Same situation—different emotions: how appraisals shape our emotions. Emotion.

[114] Gross J (1998). Antecedent- and response-focused emotion regulation: divergent consequences for experience, expression, and physiology. J Pers Soc Psychol.

[115] Davis M, Redmond E, Baraban JM (1979). Noradrenergic agonists and antagonists: effects on conditioned fear as measured by the potentiated startle paradigm. Psychopharmacology.

[116] Tijssen MAJ. Hyperekplexia. Thesis. 1997, Leiden University, The Netherlands.

[117] Nishino S, Mignot E (1997). Pharmacological aspects of human and canine narcolepsy. Prog Neurobiol.

[118] Maquet P, Peters J, Aerts J, Delfiore G, Degueldre C, Luxen A, Franck G (1996). Functional neuroanatomy of human rapid-eye-movement sleep and dreaming. Nature.

[119] Maquet P, Franck G (1997). REM sleep and amygdala. Mol Psychiatr..

[120] Hobson JA, Stickgold R, Pace-Schott EF (1998). The neuropsychology of REM sleep dreaming. NeuroReport.

[121] Fagioli I (2002). Mental activity during sleep. Sleep Med Rev.

[122] Cipolli C, Ferrara M, De Gennaro L, Plazzi G (2017). Beyond the neuropsychology of dreaming: insights into the neural basis of dreaming with new techniques of sleep recording and analysis. Sleep Med Rev.

[123] Mangiaruga A, Scarpelli S, Bartolacci C, De Gennaro L (2018). Spotlight on dream recall: the ages of dreams. Nat Sci Sleep.

[124] De Gennaro L, Cipolli C, Cherubini A, Assogna F, Cacciari C, Marzano C (2011). Amygdala and hippocampus volumetry and diffusivity in relation to dreaming. Hum Brain Mapp.

[125] De Gennaro L, Lanteri O, Piras F, Scarpelli S, Assogna F, Ferrara M, Caltagirone C, Spalletta G (2016). Dopaminergic system and dream recall: an MRI study in Parkinson’s disease patients. Hum Brain Mapp..

[126] Dopheide MM, Morgan RE, Rodvelt KR, Schachtman TR, Miller DK (2007). Modafinil evokes striatal [(3)H]dopamine release and alters the subjective properties of stimulants. Eur J Pharmacol.

[127] Qu WM, Huang ZL, Xu XH, Matsumoto N, Urade Y (2008). Dopaminergic D1 and D2 receptors are essential for the arousal effect of modafinil. J Neurosci..

[128] Madras BK, Xie Z, Lin Z, Jassen A, Panas H, Lynch L (2006). Modafinil occupies dopamine and norepinephrine transporters in vivo and modulates the transporters and trace amine activity in vitro. J Pharmacol Exp Ther.

[129] Zolkowska D, Jain R, Rothman RB, Partilla JS, Roth BL, Setola V (2009). Evidence for the involvement of dopamine transporters in behavioral stimulant effects of modafinil. J Pharmacol Exp Ther.

[130] Schredl M (1998). Dream content in patients with narcolepsy: preliminary findings. Dreaming..

[131] Bourguignon A, Guillerminault C, Dement WC, Passouant P (1976). Narcolepsy and psychoanalysis. Narcolepsy, proceedings of the first international symposium.

[132] Lee JH, Bliwise DL, Lebret-Bories E, Guilleminault C, Dement WC (1993). Dream disturbed sleep in insomnia and narcolepsy. J Nerv Ment Dis.

[133] Vogel GW (1976). Mentation reported from naps of narcoleptics. Adv Sleep Res..

[134] Fosse R, Stickgold R, Hobson JA (2002). Emotional experience during rapid-eye-movement sleep in narcolepsy. Sleep.

[135] Fosse R (2000). REM mentation in narcoleptics and normals: an empirical test of two neurocognitive theories. Conscious Cogn.

[136] Wamsley E, Donjacour CE, Scammell TE, Lammers GJ, Stickgold R (2014). Delusional confusion of dreaming and reality in narcolepsy. Sleep.

[137] Rak M, Beitinger P, Steiger A, Schredl M, Dresler M (2015). Increased lucid dreaming frequency in narcolepsy. Sleep.

[138] Dodet P, Chavez M, Leu-Semenescu S, Golmard JL, Arnulf I (2015). Lucid dreaming in narcolepsy. Sleep.

[139] Voss U, Holzmann R, Tuin I, Hobson JA (2009). Lucid dreaming: a state of conciousness with features of both waking ans non-lucid dreaming. Sleep.

[140] Meaidi A, Pourhadi N, Ptito M, Kupers R, Jennum P (2016). Dreams in narcoleptic patients with and without cataplexy. J Sleep Res..

[141] Pisko J, Pastorek L, Buskova J, Sonka K, Nevsimalova S (2014). Nightmares in narcolepsy: under investigated symptom?. Sleep Med..

[142] Li SX, Zhang B, Li AM, Wing YK (2010). Prevalence and correlates of frequent nightmares: a community-based 2-phase study. Sleep.

[143] Sandman N, Valli K, Kronholm E, Ollila HM, Revonsuo A, Laatikainen T (2013). Nightmares: prevalence among the Finnish general adult population and war veterans during 1972–2007. Sleep.

[144] Spoormaker VI, Van den Bout J, Meijer EJG (2003). Lucid dreaming treatment for nightmares: a series of cases. Dreaming.

[145] Spoormaker VI, Van den Bout J (2006). Lucid dreaming treatment for nightmares: a pilot study. Psychother Psychosom.

[146] Zadra AL, Pihl RO (1997). Lucid dreaming as a treatment for recurrent nightmares. Psychother Psychosom.

[147] LaBerge S, Phillips L, Levitan L (1994). An hour of wakefulness before morning naps makes lucidity more likely. NightLight.

[148] Stumbrys T, Erlacher D, Schädlich M, Schredl M (2012). Induction of lucid dreams: a systematic review of evidence. Conscious Cogn.

[149] Bagby RM, Parker JDA, Taylor GJ (1994). The twenty-item Toronto Alexithymia Scale–I. Item selection and cross-validation of the factor structure. J Psychosom Res..

[150] Wise TN, Mann LS, Mitchell JD (1990). Secondary alexithymia: an empirical validation. Compr Psychiatry.

[151] Yehuda R, Steiner A, Kahana B (1997). Alexithymiain Holocaust survivors with and without PTSD. J Trauma Stress.

[152] Zeitlin SB, McNally RJ, Cassiday KL (1993). Alexithymia in victims of sexual assault: an effect of repeated traumatization?. Am J Psychiatry.

[153] de Vente W, Kamphuis JH, Emmelkamp PM (2006). Alexithymia, risk factor or consequence of work-related stress?. Psychother Psychosom.

[154] Freyberger H (1977). Supportive psychotherapy techniques in primary and secondary alexithymia. Psichother Psychosom.

[155] Naumann A, Daum I (2003). Narcolepsy: pathophysiology and neuropsychological changes. Behav Neurol..

[156] Lindsley G, Goswami M, Thorpy M, Pandi-Perumal S (2016). Narcolepsy, intimacy, and sexuality. Narcolepsy.

[157] Apfel RJ, Sifneos PE (1979). Alexithymia: concept and measurement. Psychother Psychosom.

[158] Krystal H (1979). Alexithymia and psychotherapy. Am J Psychother.

[159] Nemiah JC (1977). Alexithymia: theoretical considerations. Psychother Psychosom.

[160] Sifneos PE (1973). The prevalence of ‘alexithymia’ characteristics in psychosomatic patients. Psychother Psychosom.

[161] Levitan HL (1978). The significance of certain dreams reported by psychosomatic patients. Psychother Psychosom.

[162] Warnes H (1986). Alexithymia, clinical and therapeutic aspects. Psychother Psychosom.

[163] Ouellet L, Nielsen TA, Montplaisir J, Cartier A, Malo JL, Lassonde M (1996). L’alexithymie, re´ponse affective et reˆ ves: investigation en laboratoire de trois caracte´ ristiques sous-jacentes au de´ ficit de l’expression des e´motions. Revue Internationale De Psychopathologie.

[164] Lumley MA, Bazydlo RA (2000). The relationship of alexithymia characteristics to dreaming. J Psychosom Res.

[165] De Gennaro L, Ferrara M, Cristiani R, Curcio G, Martiradonna V, Bertini M (2003). Alexithymia and dream recall upon spontaneous morning awakening. Psychosom Med.

[166] Bauermann TM, Parker JDA, Taylor GJ (2008). Sleep problems and sleep hygiene in young adults with alexithymia. Personality Individ Differ.

[167] Shin LM, Rauch SL, Pitman RK (2006). Amygdala, medial prefrontal cortex, and hippocampal function in PTSD. Ann N Y Acad Sci..

[168] Germain A, Buysse DJ, Nofzinger E (2008). Sleep-specific mechanisms underlying post traumatic stress disorder: integrative review and neurobiological hypotheses. Sleep Med Rev..

[169] Braun AR, Balkin TJ, Wesenten NJ, Carson RE, Varga M, Baldwin P, Selbie S, Belenky G, Herscovitch P (1997). Regional cerebral blood flow throughout the sleep-wake cycle. An H2(15)O PET study. Brain..

[170] Braun AR, Balkin TJ, Wesensten NJ, Gwadry F, Carson RE, Varga M, Baldwin P, Belenky G, Herscovitch P (1998). Dissociated pattern of activity in visual cortices and their projections during human rapid eye movement sleep. Science.

[171] Nofzinger EA, Mintun MA, Wiseman M, Kupfer DJ, Moore RY (1997). Forebrain activation in REM sleep: an FDG PET study. Brain Res..

[172] Maquet P (2000). Functional neuroimaging of normal human sleep by positron emission tomography. J Sleep Res.

[173] Corsi-Cabrera M, Velasco F, Del Río-Portilla Y, Armony JL, Trejo-Martínez D, Guevara MA, Velasco AL (2016). Human amygdala activation during rapid eye movements of rapid eye movement sleep: an intracranial study. J Sleep Res..

[174] Nishida M, Pearsall J, Buckner RL, Walker MP (2009). REM sleep, prefrontal theta, and the consolidation of human emotional memory. Cereb Cortex.

[175] Marzano C, Ferrara M, Mauro F, Moroni F, Gorgoni M, Tempesta D, Cipolli C, De Gennaro L (2011). Recalling and forgetting dreams: theta and alpha oscillations during sleep predict subsequent dream recall. J Neurosci..

[176] Scarpelli S, Marzano C, D’Atri A, Gorgoni M, Ferrara M, De Gennaro L (2015). State- or trait-like individual differences in dream recall: preliminary findings from a within-subjects study of multiple nap REM sleep awakenings. Front Psychol..

[177] Siclari F, Baird B, Perogamvros L, Bernardi G, LaRocque JJ, Riedner B, Boly M, Postle BR, Tononi G (2017). The neural correlates of dreaming. Nat Neurosci..

[178] Scarpelli S, D’Atri A, Mangiaruga A, Marzano C, Gorgoni M, Schiappa C, Ferrara M, De Gennaro L (2017). Predicting dream recall: EEG activation during NREM sleep or shared mechanisms with wakefulness?. Brain Topogr..

[179] Schredl M, Binder R, Feldmann S (2012). Dreaming in patients with sleep disorders: a multicenter study. Somnologie.

